# Complete Genome Sequence of Polycladomyces abyssicola JIR-001^T^, Isolated from Hemipelagic Sediment in Deep Seawater

**DOI:** 10.1128/MRA.00435-21

**Published:** 2021-06-17

**Authors:** Taishi Tsubouchi, Yukihiro Kaneko

**Affiliations:** a Department of Bacteriology, Osaka City University, Graduate School of Medicine, Abeno, Osaka, Japan; b Research Center for Infectious Disease Sciences, Osaka City University, Graduate School of Medicine, Abeno, Osaka, Japan; c Toneyama Institute for Tuberculosis Research, Osaka City University, Graduate School of Medicine, Toneyama, Toyonaka, Japan; Montana State University

## Abstract

Here, we report the complete genome sequence of Polycladomyces abyssicola strain JIR-001, which we isolated from hemipelagic sediment in deep seawater. The genome, generated by combining long-read (Flongle) and short-read (NovaSeq) sequencing data, is 3,197,230 bp, with a mean G+C content of 52.0%.

## ANNOUNCEMENT

The genus *Polycladomyces* consists of only two species at the time of writing (1, 2); nevertheless, genomic sequences of either type strain have not been analyzed. Polycladomyces abyssicola JIR-001^T^, which was isolated using BSW-1 medium from a deep-seafloor sediment subsample collected from the Shimokita Peninsula of Japan and reported in 2013 for the first time, is the type species of the genus (1). Phenotypically, *P. abyssicola* is a thermophilic, nonmotile, aerobic, and heterotrophic filamentous bacterium; however, little is known about its detailed microbial characteristics. Here, we announce the complete genome sequence of *P. abyssicola* JIR-001^T^. Strain JIR-001^T^, stored at −80°C, was streaked onto a BSW-1 agar plate (1) and incubated at 60°C for about 4 days. A 14-ml round-bottom tube containing 3 ml of BSW-1 liquid medium was inoculated with a single colony picked from this plate and incubated at 60°C and 80 rpm for about 5 days. Genomic DNA extraction was performed using the Genomic-tip 20/G kit (Qiagen). Whole-genome sequencing was performed using the MinION system (Oxford Nanopore Technologies [ONT]) with a Flongle flow cell (R9.4.1) and the NovaSeq system (Illumina).

For Illumina sequencing, the library was prepared with the TruSeq Nano kit and sequenced using paired-end 150-bp reads with a NovaSeq 6000 sequencer. Fastp v0.18.0 (3) was used to filter the raw Illumina reads. For ONT sequencing, the genomic DNA was sheared into 10-kbp fragments using a g-TUBE (Covaris), and the library was prepared using the 1D ligation sequencing kit (SQK-LSK108). In this study, when using some software, default parameters were used except where otherwise noted. Base calling of the ONT reads was performed using Guppy v3.2.1. The Flongle reads were filtered using Filtlong v0.2.0 (lengths of ≥1,000 bp and quality scores of ≥9) to keep only the top 90% of reads and with a target output of 250 Mbp (*N*_50_, 5,717 bp). These filtered ONT reads were combined with the Illumina platform and were *de novo* assembled into one contig using Unicycler v0.4.8 (4).

The complete genome resulted in 1 circular chromosome with a total length of 3,197,230 bp, with a mean G+C content of 52.0%. The quality of the assembled genome sequence was evaluated using Benchmarking Universal Single-Copy Ortholog (BUSCO) v5.1.2 (5) with the bacillales_odb10 data set. Out of 450 orthologs identified by BUSCO, 430 were located in the assembled genome sequence. A total of 3,200 coding DNA sequences (CDSs) and 13 rRNAs (4 sets of rRNA clusters and 1 additional 5S rRNA gene) were annotated by the DDBJ Fast Annotation and Submission Tool (DFAST) server (6, 7). Using Pilon (8), the *dnaA* gene on the chromosome and *rep*-like gene on the plasmid were the first genes.

### Data availability.

The complete genome sequence of *P. abyssicola* strain JIR-001^T^ has been deposited in DDBJ under the accession number AP024601. The raw sequence data are available in the Sequence Read Archive with the accession numbers DRX274628 and DRX274629.
